# Proteins Directly Interacting with Mammalian 20S Proteasomal Subunits and Ubiquitin-Independent Proteasomal Degradation

**DOI:** 10.3390/biom4041140

**Published:** 2014-12-19

**Authors:** Raúl Sánchez-Lanzas, José G. Castaño

**Affiliations:** 1 Departamento de Bioquímica, Instituto de Investigaciones Biomédicas ‘Alberto Sols’, UAM-CSIC; Facultad de Medicina de la Universidad Autónoma de Madrid, Madrid 28029, Spain; E-Mail: rslanzas@iib.uam.es; 2 Centro de Investigación Biomédica en Red sobre Enfermedades Neurodegenerativas (CIBERNED); Valderrebollo 5, Madrid 28041, Spain

**Keywords:** proteasome, proteasome interactions, ubiquitin-dependent, ubiquitin-independent, degradation, proteolysis, proteasome activators, transcription, cell cycle, neurodegeneration

## Abstract

The mammalian 20S proteasome is a heterodimeric cylindrical complex (α7β7β7α7), composed of four rings each composed of seven different α or β subunits with broad proteolytic activity. We review the mammalian proteins shown to directly interact with specific 20S proteasomal subunits and those subjected to ubiquitin-independent proteasomal degradation (UIPD). The published reports of proteins that interact with specific proteasomal subunits, and others found on interactome databases and those that are degraded by a UIPD mechanism, overlap by only a few protein members. Therefore, systematic studies of the specificity of the interactions, the elucidation of the protein regions implicated in the interactions (that may or may not be followed by degradation) and competition experiments between proteins known to interact with the same proteasomal subunit, are needed. Those studies should provide a coherent picture of the molecular mechanisms governing the interactions of cellular proteins with proteasomal subunits, and their relevance to cell proteostasis and cell functioning.

## 1. Introduction

The proteasome is a 2.5 MDa complex formed by a proteolytic core particle (20S, CP), and is a cylindrical shaped complex with a heterodimeric structure (α7β7β7α7 subunits). Attached to both bases of the cylinder is a regulatory particle (19S, RP) that consists of a horseshoe-like complex composed of a base and a lid. There are two copies of each of the three catalytic β subunits (β1, β2 and β5) in the CP and their active sites are located inside the catalytic chamber formed by the contiguous β rings [1].

The consensus mechanism of protein degradation by the 26S proteasome states that ubiquitin must be attached to the protein so that it can be tagged for degradation. The process starts with the recognition of the poly-ubiquitylated protein which is carried out by the base of the RP which leads to de-ubiquitylation (the lid of the RP), the unfolding of the protein (the base), and translocation into the catalytic chamber of the CP for proteolysis [1]. However, an increasing number of studies have proved the existence of alternative mechanisms for protein degradation by the proteasome, which do not require prior ubiquitylation. Proteins directly degraded by a ubiquitin-independent proteasomal degradation mechanism (UIPD) must belong to the large set of proteins that interact with the proteasome which include modulators or accessory proteins of proteasomal function.

Our aim is to provide a critical assessment of the research carried out in this area, by analyzing the specific interactions of mammalian cellular proteins with specific 20S (CP) proteasomal subunits. This will enable identification of the set of proteins that interact with the proteasome, and a comparison of these proteins with the set of proteins degraded by the UIPD mechanism [2]. Finally, we provide some suggestions for further research in this area.

## 2. Interaction of Cellular Proteins with Specific Proteasomal α and β Subunits of the 20S Proteasome Complex

We performed several general (alpha or beta proteasome subunits) or specific (using the acronym of each subunit) searches into the published literature to identify proteins interacting with specific CP subunits. Although we endeavoured to carry out as comprehensive a survey as possible, it may be possible that some papers have been overlooked, which we recognize poses some limitations to this work. A succinct description of those protein interacting partners of specific CP subunits, and the consequences of those interactions is given below.

### 2.1. PSMA2, C3, α2

The PSMA2 subunit of the 20S proteasome complex has been shown to directly interact with IκBα through its arm-repeats [3] likely mediating its UIPD. More recently it has been shown that calcineurin also interacts with PSMA2 and promotes the degradation of IκBα by the ubiquitin-proteasome pathway [4].

### 2.2. PSMA4, C9, α3

The PSMA4 subunit interacts with amino acids 40 to 60 of Hepatitis C virus F protein and promotes its UIPD [5].

### 2.3. PSMA7, XAPC7, α4

The PSMA7 subunit has been reported to be one of the α-subunits that interacts with the REGα/β (PA28 α/β) proteasomal activator as shown by yeast two-hybrid experiments, and the inhibition of proteasomal activation by the hepatitis B virus X protein-derived polypeptide, which binds directly to the PSMA7 subunit [6]. PSMA7 C-terminus also interacts specifically with the N-terminal region of Rab7 and participates in the late endocytic transport of cargo proteins, but this interaction does not promote Rab7 degradation [7]. Parkin, an E3 ligase implicated in Parkinson disease (PD), interacts through its C-terminus IBR-RING with the C-terminal region of PSMA7, and it may function as an accessory protein for substrate presentation to the proteasome for degradation [8]. The reported interaction of hypoxia-inducible factor-1α (HIF-1α) with PSMA7 [9] suggests that it regulates its degradation and is prevented by the direct interaction of PSMA7 with calcineurin B, this results in the inhibition of HIF-1α degradation by the proteasomal pathway [10]. In the same context, Endothelial Monocyte Activating Polypeptide-II (EMAP-II) interacts with PSMA7 after internalization, increasing the degradation of HIF-1α under hypoxic conditions [11]. Finally, PSMA7 also interacts with the nucleotide-binding oligomerization domain-containing protein 1 (NOD1) promoting its degradation by the proteasome [12].

### 2.4. PSMA3, C8, α7 Subunit

PSMA3 is known to form double ring heptameric structures (540 kDa) when expressed as a recombinant protein in bacteria [13]. PSMA3 is also able to form heterogeneous 540 kDa complexes with alphaB-crystallin, although alphaB-crystallin does not directly interact with the proteasome [14]. PSMA3 is also one of the subunits that interacts with REGα/β (PA28α/β) mediating proteasomal activation [6] together with PSMA1 [15] and PSMA7 (as described above).

Egr-1 [16] and aurora/Ipl1-related kinase 2 (Aurora-B) [17] interact with PSMA3, but it is unclear if those interactions are involved in the ubiquitin-dependent proteasomal degradation (UDPD) of Egr-1 or Aurora-B. The C-terminus of p21WAF1/CIP1 interacts with PSMA3 promoting its degradation by a UIPD mechanism [18]. Apart from this direct interaction, several proteins have been shown to mediate presentation of p21 to the proteasome complex. MDM2, an E3 ubiquitin ligase, does not ubiquitylate p21, but through the region comprising amino acids 180–298, binds to p21, enhancing the binding of p21 to the PSMA3 proteasomal subunit for UIPD of p21 [19]. 14-3-3tau protein also binds to p21, MDM2, and PSMA3, facilitating the targeting of p21 to degradation [20]. Finally, binding p21 to REGγ (PA28γ) a proteasome activator, also seems to facilitate p21 degradation by the proteasome [21,22]. Id-1 interacts with PSMA3, and this interaction seems to be critical for the degradation of the Hepatitis-B virus (HBV)-encoded protein, HBX which requires ubiquitylation to be degraded by the proteasome [23]. SRC-3/AIB1 is a steroid receptor coactivator that can interact directly with PSMA3 subunit [24] or bind to REGγ (PA28γ) for presentation to the proteasome for degradation [25]. MDM2 also binds to PSMA3 and promotes Rb-PSMA3 interaction, leading to UIPD of Rb [26,27]. PSMA3 also interacts with the Epstein-Barr virus (EBV)-encoded nuclear proteins EBNA3A, EBNA3B and EBNA3C that are directly degraded *in vitro* by the proteasome [28]. *In vitro* studies have also shown that PSMA3 interacts with splicing factors and other proteins involved in RNA metabolism [29]. Finally, the N-terminal region (amino acids 1–60) of alpha-synuclein, a protein implicated in PD, interacts with the C-terminal region of PSMA3, which is essential for its degradation by the 20S proteasome [30].

### 2.5. PSMB6, Y, β1

PSMB6 has been shown to interact with Plasminogen Activator Inhibitor-2 (PAI-2) and this interaction may mediate its anti-apoptotic role [31]. PSMB6 has also been shown to bind directly to p27Kip1 promoting its direct degradation by the proteasome [32,33].

### 2.6. PSMB1, C5, β6

The intracellular domain of TrkA has been reported to interact with several proteins, including the PSMB1 proteasomal subunit, while TrkB and TrkC do not. This TRkA interaction results in the phosphorylation of PSMB1, although with unknown consequences in proteasomal function [34].

### 2.7. PSMB4, N3, β7

HTLV-I Tax has been shown to interact with PSMB4 and may contribute to the targeting of either p105 or p65, and IκBα to the proteasome for processing or degradation, respectively [35]. Smad1 is targeted for degradation by the ubiquitin-dependent mechanism as well as by binding to PSMB4, and to ornithine decarboxylase antizyme (Az), likely to be degraded by a UIPD mechanism [36].

**Figure 1:**
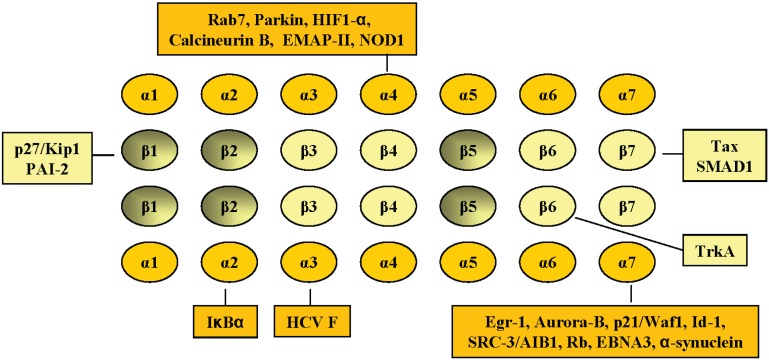
Schematic diagram of 20S proteasomal subunits and mammalian cellular proteins reported to interact with specific proteasomal subunits. The different α subunits are shaded in bright yellow and the β subunits are pale yellow. Active β subunits (β1, β2 and β5) are coloured black degraded to pale yellow. Mammalian proteins reported to interact with a specific proteasomal subunit are indicated by rectangular boxes. For further details refer to the main text.

The subunit specific interactions described above are schematically summarized in Figure 1, although this figure does not include the interaction with the proteasomal activators REGα/β (PA28 α/β) and REGγ (PA28γ).

## 3. Ubiquitin Independent Proteasomal Degradation

The list of cellular proteins whose degradation does not necessitate prior ubiquitylation is increasing. The proteins that have already been described [2,37] include: ornithine decarboxylase (ODC) either directly or mediated by the Az, p21 (see above), p53 whose degradation is inhibited by NAD(P)H:quinone oxidoreductase 1 (NQO1), c-Fos also inhibited by NQO1 [38] and Fra-1 which interacts with the19S proteasomal subunit, TBP-1 that has a TBP-1 ubiquitin-independent C-terminal degron [39], Rb presented by human cytomegalovirus pp71 protein or MDM2 (see above), alpha-synuclein (see above), HIF-1α, SRC-3/AIB1 transcriptional coactivator (see above), NF-κB p105 processing into p50 and the degradation of IκBα (see above), Y-box-binding protein 1 (YB-1), thymidylate synthase (TS) and Tau protein which is implicated in Alzheimer’s disease.

Further proteins have been added to the list of those being degraded by a UIPD mechanism since the last extensive revision [2]. The new proteins reported to be degraded (presented below) are ordered by the date of publication, and include proteins implicated in many cellular functions or pathways.

Proteins implicated in DNA and chromatin structure degraded by UIPD include BAF57, a component of the mammalian SWI/SNF chromatin remodelling complex [40], and Topoisomerase IIβ (Top2β) degraded by the 26S proteasome after RNA polymerase II blockage [41]. Transcription factors reported to be degraded by a UIDP mechanism include: KLF5 a Kruppel-like zinc finger transcription factor [42], DNp73, a transactivation-deficient and anti-apoptotic form of p73 whose degradation is mediated by Az [43], Bob1 (Obf-1 or OCA-B) a transcriptional coactivator [44] and IκBNS which acts as an inhibitor of a subset of NF-κB target genes [45].

Proteins implicated in cell cycle control and apoptosis that have been reported to be degraded by UIPD include: Aurora-A mediated by Az and regulated by binding to AURKAIP1 [46], Daxx whose degradation is promoted by human cytomegalovirus (HCMV) pp71 [47], MCL-1 anti-apoptotic myeloid cell leukemia 1 [48], BIM-extra long (BIM(EL) a pro-apoptotic BH3-only protein [49], NOXA an unstructured BH3-only protein [50], a homeodomain transcription factor NKX3.1 whose degradation is mediated by its C-terminal 21-amino acid domain [51], and nucleostemin a nucleolar GTP-binding protein essential for ribosomal biogenesis whose degradation is controlled by GTP levels [52].

Viral proteins degraded by UIPD include: HBX which probably regulates gene transcription [53], human cytomegalovirus (HCMV) pUL21 a protein required for establishing an HCMV infection [54], murine cytomegalovirus (MCMV) pM141 a protein that together with pM140 is required for virion assembly [55] and the hepatitis C virus (HCV) p7 protein, a hexameric protein forming a funnel-like structure in the membranes which play a critical role in the virion life-cycle [56].

Other proteins with diverse cellular functions reported to be degraded by UIPD include: processing of the N-terminus of LC3 an ubiquitin-like protein that plays an essential role in autophagy [57], connexin43 (Cx43) whose degradation is stimulated by CIP75 [58], RILaltCterm an alternatively spliced isoform of RIL that activates actin bundling [59], voltage-gated Kv7.2/KCNQ2/M-channel C-terminal which has a frame-shift mutation that has been found in benign familiar epilepsy [60], DJ-1 L166P a missense mutant implicated in familiar forms of PD [61] and RCHY1 whose degradation is mediated by interaction with Hoxa2 [62].

We have also added to this list those proteins that become substrates for UIPD via their interaction with proteasomal activators, mainly REGγ (PA28γ) and PA200/Blm10. We have already described that REGγ (PA28γ) can present p21, but it can also present other cell cycle regulators such as p16 (INK4A) and p19 (Arf) to proteasomes for degradation [21,22] and SRC-3 a coactivator for UIPD [25]. The levels of activation-induced deaminase (AID), responsible for the initiation of antibody gene diversification in activated B lymphocytes, are subjected to UIPD by interaction with REGγ (PA28γ) [19,63]. Finally, MAFA, a basic leucine zipper transcription factor implicated in insulin gene transcriptional regulation, interacts with REGγ (PA28γ) for proteasomal degradation; this interaction is dependent on MAFA phosphorylation by GSK-3 [64]. REGγ (PA28γ) also seems also to facilitate the interaction of p53 and MDM2, but in this case it promotes MDM2-dependent UDPD of p53 [65] and participates in the mechanism of the regulation of HCV core proteins, nuclear retention and degradation [66,67]. The PA200/Blm10 proteasome activator binds to the CP by its C-terminal YYX motif and activates *in vitro* degradation of tau [68] which is known to be mediated by the 20S proteasome [69]. More recently it has been shown that PA200/Blm10 promotes the UIPD of acetylated core histones by binding to the bromodomain-like regions of PA200 [70].

**Figure 2:**
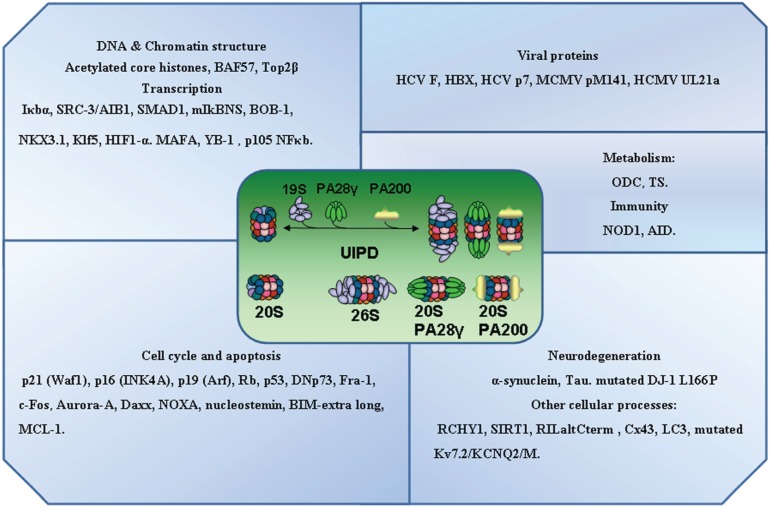
Classification of mammalian proteins reported to be degraded by an ubiquitin-independent proteasomal pathway. The protein function or cellular processes in which those proteins are involved have been used to group the different mammalian proteins that have been shown to be subjected to Ubiquitin-Independent Proteasomal Degradations (UIPD) by the 20S or 26S proteasome, or facilitated by activators (PA28γ and PA200) of the proteasome. A full description and details of the individual proteins and their UIPD mechanism may be found in the main text.

The proteins shown to be degraded by a UIPD mechanism are summarized in Figure 2, they have been classified according to the function or the cellular process in which they are involved. Many of the proteins involved in transcription, cell cycle and apoptosis, and which are also degraded by a UDPD mechanism, appear prominently as UIPD substrates. However, this fact may only be a reflection of the active research bias in these areas as demonstrated by the large number of PubMed entries retrieved using those keywords in a search.

## 4. A Critical Assessment of Specific Protein Interactions of Proteasomal Subunits and UIPD

Proteins shown to interact directly with the different mammalian 20S proteasomal subunits and which do not belong to the RP complex are summarized in Figure 1. Those proteins constitute a set which are not all degraded by a UIPD mechanism (Figure 2). Many more proteasomal interacting proteins can be found in interactome databases. For example, PSMA3 in the BIOGRID interactome database is reported to interact with 148 different cellular proteins without taking into account those protein partners that are components of the proteasomal CP or RP. The protein-partners of PSMA3 shown in Figure 1 are included in interactomic databases. In general, the high-throughput methods generate interactome data sets that still have numerous false positives; therefore further experimental evidence is needed to ascertain the relevance of those interactions.

Proteasome-like structures are as old in evolutionary terms, as C-terminal diglycine ubiquitin or ubiquitin-like molecules, and are unevenly distributed in the different kingdoms including *Archea* [71]. If the unique function of the simple *Archea* proteasome, formed by only one (or two) type(s) of α and β subunits, was protein degradation, this would suggest the existence of an archaic macromolecular complex with hidden catalytic sites which would offer two types of subunits for interactions with “thousands” of protein substrates for degradation or other functional consequences. What is the molecular basis of those interactions? Certainly, our knowledge is very limited at present. Duplication of the α and β genes, and diversification of their sequences has occurred during evolution [71]. In order to maintain the functional structure of the proteasome, those sequence changes must occur in regions that are not relevant, or that are compensated by changes in the corresponding interacting subunits of the proteasome complex. This would be necessary to keep the basic cylindrical structure and the correct processing of the active pre-β subunits. In eukaryotes, which have fourteen types of CP proteasomal subunits, the number of cellular protein interacting partners is likely to have increased. What are the specific sequences and structural determinants responsible for the presumed increase in the number of interacting proteins? The answer may be provided by studies of the proteins that interact with archeal proteasomal subunits together with high-resolution X-ray data of this proteasome and the proteasome-cellular protein complexes (which would probably also be feasible for yeast proteasomes). The results of these investigations may eventually provide the atomic details of the binding site locations and the physicochemical properties of the interaction interfaces. These studies could also provide us with an evolutionary perspective, and would certainly help discover the basic ‘highly’ conserved principles of the interactions between cellular proteins and proteasomal subunits, as well as providing a better understanding of the UIPD mechanism.

To validate a direct interaction between a specific proteasomal subunit and a specific cellular protein, most of the published research relies on two-hybrid studies and affinity-capture followed by mass spectrometry. Another experimental approach is the immunoblotting of pull-down experiments of crude cellular extracts, cell-free translation products or purified recombinant proteins using antibodies or recombinant proteins. Specificity, when analyzed, is defined by the use of proteasomal subunits that do not interact with the protein under study, or with a structural modification of the proteasomal subunit and/or the corresponding interacting protein partner. Those structure variants allow the determination of which regions of both partners are involved in the interaction. Alternatively, some groups have used cells expressing tagged proteasomal subunits to explore the binding of endogenous, or transfected tagged, cellular protein partners by co-immunoprecipitation or binding to affinity-resins.

A critical analysis of the *in vitro* experiments reveals that the reported interactions that either cannot be reproduced using the entire proteasomal complex (because the structure and the surface offered by the proteasomal subunits in the complex differs from the unassembled proteasomal subunits) or, where the interaction cannot be demonstrated in cell lysates, are irrelevant. This criticism would be correct for those interacting protein partners that are not directly degraded by the proteasome, but would not be sustainable for those that are degraded by the proteasome. In this case, the productive interaction ends in an enzymatic reaction. Accordingly, it may be difficult to isolate the CP-interacting protein intermediate of the reaction even at lower temperatures, or in the presence of proteasome inhibitors. The binding energy may contribute to lowering the energy barrier of peptide bond hydrolysis by an induced conformational change of the proteasome and/or the active sites. The alternative to validate the reported interaction would be through kinetic competition experiments, using specific inhibition of the degradation of the interacting protein by constructs of the specific proteasomal subunit *in vitro or* by transfection in cells, provided that it is demonstrated that the proteasome complex structure is not affected. Furthermore, competition experiments using proteins that are reported to bind to the same (or different, allowing specificity of the competition to be studied) proteasomal subunits would be very helpful to understand the relative kinetic constants and the strength of the interactions between the different proteins.

The same experimental approach could also be applied to cell studies albeit with some obvious limitations. One clear limitation to the analysis of the interactions with proteasome subunits in the cell would be that the amount of free and unassembled 20S proteasomal subunits is likely to be very low, with the possible exception of tumor cells where they are overproduced and degraded [72,73]. Competition experiments by over expression of a proteasomal subunit (untagged or tagged in the C-terminus) will displace the corresponding endogenous subunit and assemble instead in the newly synthesized and assembled proteasome. Unless the transfected proteasomal subunit remains unassembled in the cell, it would be difficult to validate these experiments as proof of the specific interaction of a protein with a specific proteasomal subunit. The same applies to the interruption of the expression of proteasomal subunits by sh or siRNA interference. The time required to downregulate the quantity of one or several subunits from the ‘old, fully assembled’ and pre-existing 20S complex is dependent on the half-life of the mature 20S complex, which is estimated to be more than a week [74]. In view of these caveats, one clear way to demonstrate the relevance *in vivo* of a specific interaction found *in vitro* would be to demonstrate the competition between two protein partners that bind to the same proteasomal subunit.

The number of proteins which participate in many cellular functions (Figure 2), described as being degraded by a UIPD mechanism is clearly increasing. It has been estimated that approximately 20% of total cell protein could be degraded by a UIPD mechanism [75]. The criteria used to establish that a protein is degraded by this mechanism are: the Lys-less version of the protein substrate (all Lys mutated to Arg) must be degraded by the proteasome, and blocking the N-terminal Met either chemically or with a tag, should not affect the degradation of the Lys-less protein by the proteasome. The above criteria exclude both internal Lys and N-terminal Met ubiquitylation establishing a UIPD mechanism for that particular protein. An exception would be those proteins that may not require Lys or Met for ubiquitin conjugation and degradation, but N-terminal acetylation [76,77] by a UIPD mechanism. To our knowledge, no such proteins have been reported. Not all the proteins reported as degraded by a UIPD mechanism (described above and summarized in Figure 2) fully satisfy the criteria mentioned above. Furthermore, many of those reported to be degraded by UIPD can also be degraded by a UDPD mechanism. The relevance or the significance of the existence of two mechanisms (UIPD and UDPD) for the same cellular protein is unclear. We could speculate that UIPD may be a default proteostatic mechanism, while UDPD a fast-adaptive response to control the proteostasis of those proteins.

There have been some attempts to determine the minimal requirements of a protein substrate to be degraded by a UIPD mechanism [78,79,80], but its generalization is unclear. The consensus is that many of the proteins degraded by UIPD mechanism have *in toto* (p21, α-synuclein, Tau) or in part (p53, HIF1-α) of its sequence, a so-called unstructured region [81,82]. Energetically, it can be conceived that those regions will facilitate their binding and translocation in the interior of the catalytic chamber of the proteasome. However, the specific and non-specific interactions with proteasomal subunits that mediate the process need to be defined. A critical issue is the determination of which of the proteasomal subunits, of both the 20S or 19S complexes, specifically interact with those proteins reported to be degraded by a UIPD mechanism.

Finally, another criticism commonly made in the reports of the UIPD of a protein substrate is that the 19S complex or proteasomal activators have to participate in this process. This is because the α-ring channel of the CP is too narrow and has to be opened to allow the transit of the extended protein into the catalytic chamber where it will be degraded [1]. As a consequence of this principle, the CP alone is inactive, except for small peptides that may diffuse freely. It is not easy to answer the question about whether the 20S proteasome has proteolyitc activity or only peptidase activity. Probably, only NMR studies will be able to unambiguously answer that question. In the meantime, it would be useful to think that proteasomes may not only behave as top-down degrading nano-cylinders (the predominant vision nowadays), but may also be lateral degrading nano-cylinders, like a lawn-mower (Figure 2, central diagram). Broadening the paradigm, protein substrates may also access the catalytic chamber of the CP through the space between the α and β rings of the cylinder. This heterodox hypothesis has already been postulated when the crystal structure of the yeast 20S proteasome was reported [83].

## 5. Conclusions

Proteasome subunits have been shown to interact with many cellular proteins; we have only described those, reported in specific published research papers, which interact with mammalian CP subunits. It is time to start to qualify the specificity, the regions of both partners implicated in the process, and to quantify, using competition experiments, the relative strength of the interactions of those proteins, both *in vitro* and *in vivo*. The degradation of cellular proteins by a UIPD mechanism implies the binding, either directly or indirectly (via another protein interacting with the proteasome) of those proteins to proteasomal subunits. It is also time to determine the proteasomal subunits responsible for this UIPD mechanism. Again, specificity, the protein regions implicated, and competition experiments will contribute to a better understanding of the mechanism of their proteasomal degradation. Both approaches could combine to form a coherent picture of the relevance of the cellular protein interactions with the proteasome and proteasomal function (including the UIDP mechanism) in cell proteostasis and cell function. Further investigations of the molecular basis of the UIDP and UDPD mechanism of the same proteins, where applicable, and those that are only degraded by a UDPD mechanism, would provide a relational and hierarchical view of the proteasome pathway, as the main proteostatic mechanism of protein degradation in the cell.
